# Nutrition, BMI and Motor Competence in Children with Autism Spectrum Disorder

**DOI:** 10.3390/medicina55050135

**Published:** 2019-05-15

**Authors:** Ting Liu, Julie Kelly, Lyndsay Davis, Krystal Zamora

**Affiliations:** Department of Health and Human Performance, Texas State University, San Marcos, TX 78666, USA; jkelly@saffairs.msstate.edu (J.K.); lyndsaydavis1239@gmail.com (L.D.); zkrystal73@gmail.com (K.Z.)

**Keywords:** autism, children, nutrition knowledge, body weight, developmental disorder

## Abstract

*Background and objectives:* The purpose of this study was to examine the relationship between motor competence, body mass index (BMI), and nutrition knowledge in children with autism spectrum disorder (ASD). *Materials and Methods:* Fifty-one children with ASD (five females and 46 males) aged 7–12 participated in the study. The Movement Assessment Battery for Children-2 (MABC-2) was used to examine children’s fine and gross motor skill competence; the nutrition knowledge survey assessed children’s overall knowledge of food groups and healthful eating; and BMI-for-age determined their weight status. Descriptive analysis and Pearson correlation was used to analyze the relationship between nutrition knowledge, BMI, and motor competence in children with ASD. *Results:* The majority of children with ASD (82%) showed significant motor delays in MABC-2 assessments. The BMI-for-age percentile data suggested that 20% of participants were obese, 17% were overweight, and 12% were underweight. The nutrition knowledge data indicated that 55% of children scored below 70% on accuracy in the nutrition knowledge survey. Pearson correlation analysis revealed a significant positive relationship between MABC-2 manual dexterity and nutrition knowledge (r = 0.327, *p* < 0.01), and between MABC-2 balance skills and nutrition knowledge (r = 0.413, *p* < 0.01). A significant negative relationship was also found between BMI and MABC-2 balance skills (r = −0.325, *p* < 0.01). *Conclusions:* The findings of the study suggest that nutrition knowledge and motor competence may be key factors influencing BMI in children with ASD and therefore interventions tackling both sides of the energy balance equation are necessary.

## 1. Introduction

Children with autism spectrum disorder (ASD) having a high obesity rate may result from a combination of poor nutrition knowledge and low physical activity levels. In the United States, approximately 32% of children and adolescents are overweight or obese [1]. While these numbers are alarming and have become a public health concern, it is an even greater concern for children with disabilities who have a higher incidence of being overweight and are twice as likely to be physically inactive as their peers without disabilities [2,3]. Among children with disabilities, it is reported that those with ASD have a much higher prevalence of obesity than those without ASD [4,5,6,7,8]. Specifically, Curtin et al. (2010) reported that the prevalence of obesity in children with ASD was 28.8% higher when compared to children without ASD [4]. In addition, Phillips et al. (2014) stated that the prevalence of obesity was two times higher among children with ASD than that of children with other developmental and physical disabilities [7]. They suggest that dietary behavior patterns may predispose children with ASD to even greater risk of obesity.

Children with ASD exhibit higher levels of food selectivity (i.e., only eating a narrow array of foods) than their peers and their food choices are influenced by texture, color, and smell [9,10,11,12,13]. One study reported that some children with ASD may only eat high calorie foods such as chicken nuggets and French fries because of the yellow color [12]. Other studies comparing children with ASD and typically developing children have indicated that children with ASD are “picky eaters” and consume significantly fewer fruits and vegetables and more servings of sugared beverages, both of which have been shown to increase obesity [14,15,16,17]. Furthermore, children with ASD experience more barriers to physical activity than the general population due to physical and developmental disabilities. As a consequence, they are more likely to be sedentary which results in less energy expenditure and increasing potential for excess weight [18,19,20]. Therefore, children with ASD may face bigger challenges to combat overweight and obesity than children without disabilities due to their limited physical activity and nutritional patterns that are needed to promote health and wellness.

With the rising prevalence of ASD from 1 in 88 children in 2008 to 1 in 60 children in 2018, the co-occurrence of ASD and obesity constitutes dually increasing public health concerns. The myriad of health risks associated with obesity in children with ASD make it critical to find solutions. The increasing prevalence of ASD has made developing appropriate and effective interventions to improve the health and wellness of children with ASD a high priority for researchers and practitioners [20]. Identifying possible contributing factors to the increasing rates of obesity in children with ASD is the first step to prevention. Physical activity and nutrition play important roles in childhood obesity according to the obesity definition (i.e., excess adipose tissue due to energy intake vs. energy output).

Fundamental motor skill performance is a key factor that influences children’s physical activity participation and weight status [21]. On the other hand, weight status has been shown to have a negative impact in children’s competence in performing fundamental motor skills [21,22,23]. Several studies on physical activity have reported that children with ASD are less likely to be involved in moderate to vigorous exercise and are more likely to be sedentary compared to the children without disabilities [24,25,26]. It has been well documented that children with ASD exhibit developmental delays in fine and gross motor skills leading to poor motor coordination, postural control, and balance [27,28,29,30]. As a result, children with ASD may be excluded from participation in sports and recreational games that demand more refined motor skills and therefore they are likely to be inactive. The consequence is more time spent in sedentary behaviors [31] which have been shown to increase the risk of obesity, [32,33] feelings of depression, low self-esteem, isolation and possibly overeating [34,35,36].

Understanding fundamental fine and gross motor skill performance in children with ASD is essential to overcome barriers for physical activity (i.e., less energy output) and to reduce excess weight gain. There are number of standardized tests that examine motor competence in children with ASD. It is important to choose an assessment that targets the age group of interest and the specific motor skills being examined. The Movement Assessment Battery for Children-2 (MABC-2) is a reliable and valid assessment used to test fundamental motor skills in children with ASD [37] The strength of the MABC-2 is that it tests both fine and gross motor skills such as manual dexterity, ball skills, along with static and dynamic balance. These skills are not only critical for participation in sports and recreational games, but also needed in the learning and acquisition of handwriting skills. In addition, manual dexterity has been closely linked to academic achievement [38,39]. For example, Dinehart and Manfra (2013) examined whether the fine motor skills of preschool students predict later academic performance in second grade. Results indicated that performance on both fine motor writing and object manipulation tasks had significant effects on second grade reading and math achievement [39].

Nutrition knowledge is another major factor in childhood obesity and becomes more crucial to obesity prevention along with co-occurring motor delays. Prior research indicates that nutrition knowledge is associated with weight loss [40,41,42,43,44]. Klohe-Lehman et al. (2006) examined the association of nutrition knowledge and the gained nutrition knowledge in promoting more weight loss in 141 low-income obese and overweight mothers. After an eight-week intervention emphasizing nutrition knowledge, the participants who had lost weight (≥2.27 kg) showed greater nutrition knowledge than those who had not lost weight. In addition, Triches and Giugliani (2005) investigated the association between obesity, eating habits, and nutrition knowledge in 573 school children (aged 8–10 years) [43]. They reported that childhood obesity was associated with limited nutrition knowledge and unhealthy eating habits and these children were five times more likely to be obese.

These findings have major consequences for children with ASD as they may not understand the serious health problems associated with lack of physical activity, excess body weight, and poor nutrition [45]. Moreover, children with ASD may not have the nutrition knowledge to make conscious decisions on healthy eating choices, and this may predispose them for becoming obese [20,36]. Environmental factors such as the eating habits of parents, mealtime practices, and food/snacks used as a reinforcing reward may also contribute to the problem related to the nutritional status in children with ASD [20,46]. Additionally, evidence suggests that children who are overweight or obese are more likely to be obese adults, which increases their risk for chronic diseases such as heart disease, stroke, diabetes, metabolic syndrome, and certain cancers [46,47,48,49]. As a result, the comorbidities associated with obesity along with the increased risk of becoming obese are a potential threat to the health and well-being of children with ASD [46]. Current literature on health education and awareness has mostly focused on children without disabilities [20]. There have been no reports in the literature regarding the combination effects of nutrition knowledge and motor competence in children with ASD. Therefore, the purpose of this study was to examine the effects of children with ASD’s motor competence and nutrition knowledge on BMI. It was hypothesized that BMI would be negatively related to motor competence and nutrition knowledge.

## 2. Materials and Methods

### 2.1. Participants

A sample of 51 children with ASD aged 7–12 (5 females, 46 males) were recruited through advertisements and local schools to participate in this study. The study consisted of primarily male participants because ASD is four times more common in males than females [50]. Inclusion criteria were (a) the child was diagnosed with ASD by a physician or a licensed psychologist according to the Diagnostic and Statistical Manual of Mental Disorders, fifth edition, [51]; (b) ability to understand and follow the test commands; (c) ability to communicate with the test administers. The study was approved by the University Institutional Review Board and parental consent was obtained from all the participants before any study procedures began.

### 2.2. Instrument

#### The Movement Assessment Battery for Children-Second Edition (MABC-2)

The MABC-2 is a standardized measure of motor impairment for children aged 3–16 years [37]. Its purpose is to identify and describe a child’s level of motor functioning relative to the age-matched norms. The MABC-2 measures both fine and gross motor competence skills in 3 age bands (3–6 years, 7–10 years, and 11–16 years). It involves a total of eight tasks in 3 components consisting of manual dexterity, ball skills, and balance. Raw scores are collected for each task and then converted to standard scores. Standard scores are calculated for each of the 3 components and then calculated by summing the eight tasks and converting it to a total test score. The total test score is then converted to percentiles found in the MABC-2 manual norm tables. The percentile can be interpreted in terms of a “traffic light” system: at or below 5th percentile (red zone) indicates significant movement difficulty; 6th–15th percentile (amber zone) falls in the “at risk” of having a movement difficulty; and above 15th percentile (green zone) indicates no movement difficulty detected [37].

### 2.3. The Nutrition Knowledge Survey

The nutrition knowledge survey is an assessment of nutrition knowledge for elementary school children [52]. It was adopted by Gower et al. (2010) to evaluate the impact of the “Fit Kids ‘r’ Healthy Kids” nutrition intervention targeting first through fourth grade students’ nutrition knowledge in a metropolitan area school in Salt Lake City, UT, USA. This 15-multiple-choice question survey assesses nutrition knowledge in the following domains: food groups, healthful foods, and food functions. Scores are calculated by summing the total number of correct answers and dividing by 15 to yield percent correct score. A higher percent correct score indicates better nutrition knowledge on healthy eating.

### 2.4. Body Mass Index

The most common method used to classify obesity is body mass index (BMI), which is calculated as weight in kilograms divided by the height squared in meters (kg/m^2^). For children, BMI-for-age percentiles are calculated based on the BMI-for-age growth chart [53]. Children with BMI-for-age percentile ≥ the 85th percentile are considered overweight and children with BMI for age ≥95th percentile are considered obese [54].

### 2.5. Procedures

Children’s height and weight data were collected for BMI. They were measured in light clothes and no shoes. Height was measured in inches utilizing a tape measure. Weight was measured in pounds utilizing the Health-O-Meter professional portable scale (Model 349KLX).

The Nutrition Knowledge Survey was then administered in a private classroom to minimize distraction. All questions were read aloud by the research assistant. Each question had three pictures associated with the question and the child was asked to point to the picture they thought was the correct answer. For example, the child was asked “look at the three pictures and pick the food from the fruit group” or “pick the healthiest snack.” If the child replied “I don’t know” or picked the wrong answer (i.e., chose ice cream over other choices of carrots or pointed to a cake for healthiest snack), he/she was given a zero. All questions answered correctly in each domain were totaled and divided by the total number of questions.

The MABC-2 was administered after each participant completed the nutrition knowledge survey. All sessions were videotaped to ensure the accuracy of the test administration. Investigators and research assistants followed guidelines from the MABC-2 Manual for administration of all tasks. In order to ensure inter-rater reliability, research assistants went through an extensive training prior to administering the tests. This training involved an introduction and education on the assessments followed by hands-on practice. Research assistants performed the skills on children for practice and were observed by the principal investigator to ensure proper administration and scoring of testing. Additionally, assessment scores calculated by the research assistants were compared to the principal investigator’s scores. If 90% or higher agreement was reached, research assistants were considered trained and ready to complete actual testing. Detailed verbal descriptions and clear demonstrations were given to children prior to their motor skill assessment. If a child performed incorrectly during the practice trial, additional instructions were provided to ensure the child’s proper task performance. The inter–rater agreement between the principal investigator and the research assistants was 97%. Each child received verbal description and an accurate demonstration of the task prior to testing. A practice trial was provided to assure the child understood the task. An additional demonstration was provided if the child did not respond or did the task incorrectly. If the child’s first performance trial did not meet the specific performance criterion, a second trial was administered. Each performance was scored according to the MABC-2 norms.

### 2.6. Data Analysis

Descriptive analysis was used to analyze data on nutrition knowledge, BMI, and the MABC-2. BMI and nutrition knowledge percentile scores were calculated using the standardized procedures. Pearson correlation was used to analyze the relationship between the motor competence and BMI, and the relationship between the nutrition knowledge and BMI. Correlations were also used to test the relationships between BMI and each of the 3 nutrition domains. Results were considered significant when the *p*-value was less than 0.05.

### 2.7. Ethical Approval

All procedures performed in studies involving human participants were in accordance with the ethical standards of the institutional and/or national research committee and with the 1964 Helsinki declaration and its later amendments or comparable ethical standards (IRB#2017623). Informed consent was obtained from all individual participants included in the study.

## 3. Results

The majority of children (90%) were classified in the red or amber zone in the MABC-2. More specifically, 82% of children were classified in the red zone indicating significant motor delays, 8% were classified in the amber zone demonstrating that they were at risk for motor delays, and 10% were classified in the green zone suggesting no motor delays or difficulties.

The BMI-for-age percentile data suggested that 37% of participants were classified as obese or overweight. Specifically, 20% were classified as obese, 17% were classified as overweight, and 12% were underweight. The nutrition knowledge accuracy data conveyed that 55% of participants scored below 70%, 20% scored between 70 and 89%, 10% scored 90% or higher, and 16% of participants scored a perfect score.

**Figure 1:**
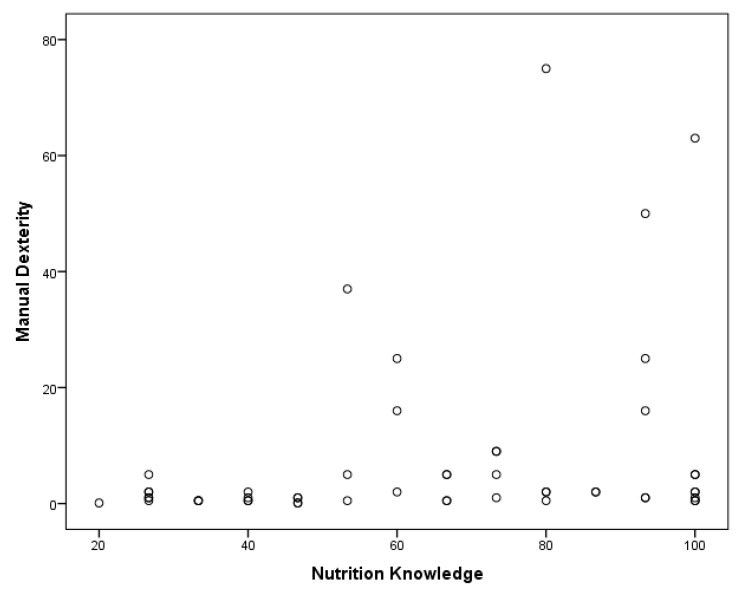
Significant positive relationship between nutrition knowledge and manual dexterity.

**Figure 2:**
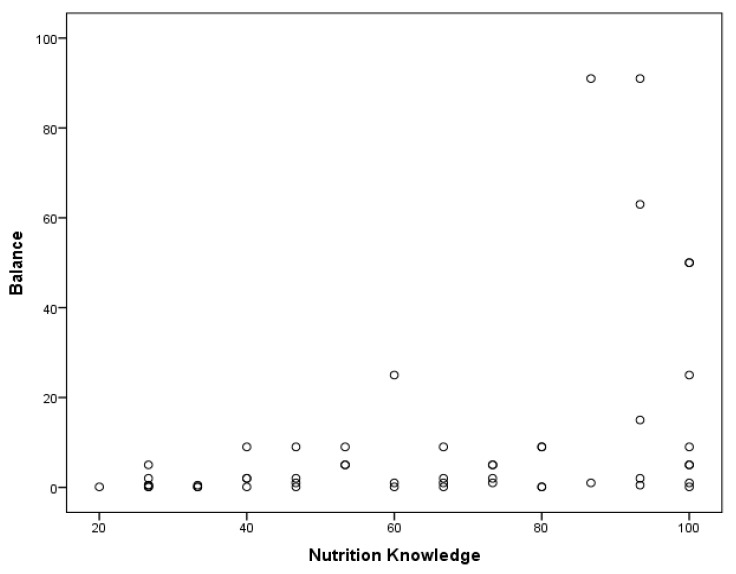
Significant positive correlation between nutrition knowledge and balance.

**Figure 3:**
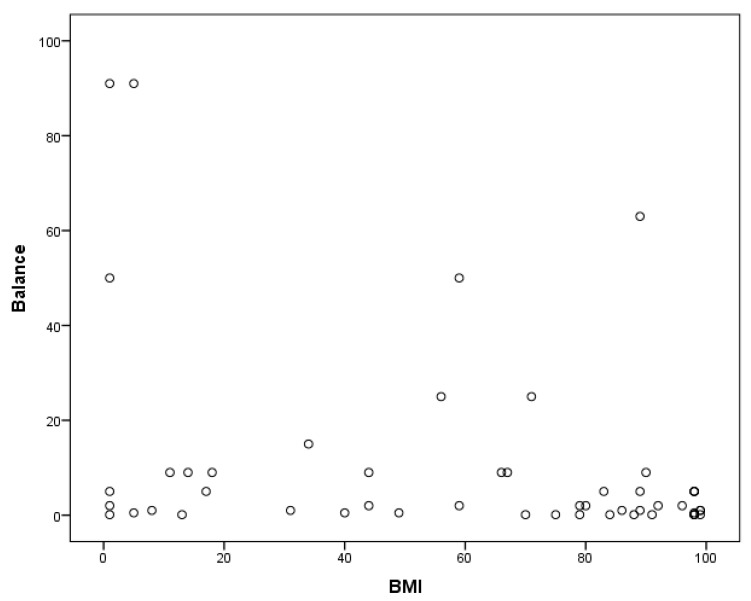
Significant negative relationship between body mass index (BMI) and balance.

A Pearson correlation coefficient was calculated for the relationship between participants’ nutrition knowledge, BMI, and motor proficiency. A significant positive correlation was found between MABC-2 manual dexterity and nutrition knowledge in children with ASD, r(49) = 0.279, *p* < 0.01 (Figure 1). Children’s static and dynamic balance were also significantly correlated with their nutrition knowledge, r(49) = 0.413, *p* < 0.01 (Figure 2). Furthermore, a significant negative relationship was found between BMI and balance, r(49) = −0.325, *p* < 0.01 (Figure 3). A regression analysis revealed that a significant regression equation was found using manual dexterity and balance to predict nutrition, *F*(2, 48) = 5.385, *p* < 0.05, with a R of 0.428 (Table 1). In addition, a significant regression equation was found between balance and BMI, *F*(1, 49) = 5.773, *p* < 0.05, with a R of 0.325 (Table 2).

## 4. Discussion

### 4.1. Nutrition Knowledge and Motor Competence

The purpose of this study was to investigate the relationship of nutrition, BMI and motor competence in children with ASD. A moderate correlation was found (r = 0.428) between nutrition knowledge and motor competence in children with ASD. Notably, 40% of the children that scored the highest on motor competence also scored the highest in nutrition knowledge. Among these participants, none were obese, two were overweight and two were healthy weight. All of the children that scored below the 25th percentile in motor competence also scored low on nutrition knowledge suggesting nutrition knowledge may be related to children’s motor competence.

One limitation of this study is that the nutrition knowledge assessment did not include dietary intake or patterns of the children and that may have been a defining characteristic of the positive correlation found between BMI and nutrition knowledge. In addition, due to the low ratio of girls to boys in children with ASD, there were only five girls that participated in this study which may have led to sample bias. Future research should examine the dietary patterns of children with ASD and include more females and the full range of children with ASD.

### 4.2. BMI and Motor Competence

A significant negative correlation was found between BMI and motor competence (r = −0.325) which supported our hypothesis. This finding is consistent with previous literature reporting similar results in children without disabilities [54,55,56,57,58]. Specifically, D’Hondt et al. (2014) reported that the children who were overweight and obese had lower motor competence scores (70.8%), particularly in physical properties requiring dynamic body coordination [58]. Overweight and obese children in the 10–12 years old group showed significantly lower motor coordination performance compared with corresponding 5–7 years old group. This finding suggested that BMI-related differences in gross motor coordination were more pronounced in older children than younger children. However, there is some discordance in data related to this topic in other studies for children with disabilities. For example, Frey and Chow (2006) examined the relationship between BMI, physical fitness, and motor skills in 444 youth aged 6–18 years with mild intellectual disabilities [59]. Consistent with the present study, Frey and colleague reported that BMI had a small, negative influence on aerobic performance (r = −0.27) and muscular strength (r = −0.18). In contrast, Pitetti, Yarmer, and Fernhall (2001) compared aerobic fitness and BMI between children aged 8–18 years with and without mild mental retardation and reported no association between BMI and their aerobic performance [60]. The discordance may be due to the differences in methodologies and more research is needed.

### 4.3. Nutrition Knowledge and BMI

The results of the present study indicated a weak correlation (r = 0.155) between BMI and nutrition knowledge suggesting that nutrition knowledge was not necessarily related to obesity in children with ASD. These findings are consistent with previous research in the literature that nutrition knowledge does not differ between obese and nonobese individuals [61,62,63,64,65]. Nutrition knowledge alone does not appear to be sufficient to maintain optimal body mass for children with ASD. Environmental factors such as family dynamics (e.g., parent’s supervision and preferences for energy-dense diets), the common use of food when children are feeling sad or lonely, or food used to reinforce good behavior may also play a role [20,66].

In contrast, other research reported a strong relationship between BMI and nutrition knowledge [67,68,69]. Yu et al. (2010) investigated the effects of nutrition education on food habit, eating behaviors, dietary attitude, nutrition knowledge, and nutrient intake in 103 overweight and obese children in the Chonbuk area [69]. Results included positive changes in the dietary attitude and nutrition knowledge, but there were no significant differences after the program suggesting nutrition education must be continued for positive food habit changes to occur in the long term. In addition, several previous studies incorporated an exercise component in the nutrition education intervention. For example, Hinckson et al. examined the effectiveness of a 10-week intervention program on physical activity, dietary habits, and overall health in children and youth aged 10–18 years with intellectual disability and autism [68]. The authors observed a trend toward reduction in BMI and waist circumference post physical activity program but by week 24, these changes were not maintained and were trivial. However, some changes in eating behavior were maintained after week 24 with the most notable being the marked reduction in consumption of confectionery sugar and chocolates. As noted, research suggests that children with ASD demonstrate problematic feeding behaviors and have higher levels of food selectivity indicating feeding problems are complex and often multifactorial. Since children’s dietary patterns evolve within the context of family, parent-child intake patterns, and preferences, they are likely to be influenced by the environmental factors such as parent nutrition knowledge, the types of foods parents make available to children at home, parental modeling of particular eating behaviors, and parent child-feeding practices [70]. Therefore, an interdisciplinary team approach may be needed that includes parents, caregivers, a registered dietician, an occupational therapist, and a behavioral psychologist to help children with ASD improve their eating and feeding behaviors and reduce their BMI in the future study [71].

## 5. Conclusions

This study has several important implications. First, although the children with ASD in this study reported having the nutrition knowledge of what to eat that is healthy, BMI scores suggest that they are not practicing this knowledge. Therefore, it is important to identify the key factors that may be influencing higher levels of obesity in this population. A thorough understanding of environmental factors including family dynamics associated with obesity are critical areas of future research needed in order to design the most effective intervention strategies. In addition, the present study suggests nutrition knowledge is moderately related to motor competence and BMI is negatively associated with motor competence. Taken together, this suggests nutrition knowledge and motor competence may be key factors influencing BMI and therefore interventions tackling both sides of the energy balance equation are necessary at both the personal and environmental level. The findings of this study were also in line with the previous research suggesting diet had a strong correlation with gastrointestinal problems in children with ASD and dietary interventions were needed to improve children’s overall health and wellbeing [72]. The literature suggests multifactorial programs promoting physical activity, nutritious diet, lifestyle education, and parental education are more effective in addressing obesity than programs concentrating on a single component such as diet or exercise [66]. Therefore, an effective treatment strategy should involve a team of experts (physicians, physical therapists, occupational therapists, behavioral therapists, nutrition and physical education experts) collaborating among the families, care givers, and special educators.

Furthermore, the majority of research has focused mostly on children without disabilities leaving a void in the literature on successful interventions for children with ASD. This void limits opportunity to reduce health risks associated with obesity in this population. We recognize that obesity is a complex problem and therefore effective health promotion interventions are needed that address the physical, cognitive, and/or sensory limitations of children with ASD and tailored to their ability and interest level [20]. Weight management among children with ASD need to focus on prevention strategies that begin early in life so that healthy lifestyle habits are established during critical developmental periods [72].

## Figures and Tables

**Table 1:** Regression analysis on manual dexterity, balance, and nutrition.

Model	Sum of Squares	df	Mean Square	F	Sig.
Regression	6250.313	2	3125.157	5.385	0.008
Residual	27,854.028	48	580.292		
Total	34,104.341	50			

Note: df= degrees of freedom; sig. = significant level.

**Table 2:** Regression analysis on balance and body mass index (BMI).

Model	Sum of Squares	df	Mean Square	F	Sig.
Regression	6815.148	1	6815.148	5.773	0.020
Residual	57,843.833	49	1180.486		
Total	64,658.980	50			

## References

[1] Ogden C.L., Carroll M.D., Kit B.K., Flegal K.M. (2014). Prevalence of childhood and adult obesity in the United States, 2011–2012. J. Am. Med. Assoc..

[2] Macdonald M., Esposito P., Ulrich D. (2011). The physical activity patterns of children with autism. BMC Res. Notes.

[3] Memari A.H., Ghaheri B., Ziaee V., Kordi R., Hafizi S., Moshayedi P. (2013). Physical activity in children and adolescents with autism assessed by triaxial accelerometry. Pediat. Obes..

[4] Curtin C., Anderson S.E., Must A., Bandini L. (2010). The prevalence of obesity in children with autism: A secondary data analysis using nationally representative data from the national survey of children’s health. BMC Pediat..

[5] Egan A.M., Dreyer M.L., Odar C.C., Beckwith M., Garrison C.B. (2013). Obesity in young children with autism spectrum disorders: Prevalence and associated factors. Child. Obes..

[6] Strahan B.E., Elder J.H. (2013). Obesity in adolescents with autism spectrum disorders. Res. Autism Spectr. Disord..

[7] Phillips K.L., Schieve L.A., Visser S., Boulet S., Sharma A.J., Kogan M.D., Yeargin-Allsopp M. (2014). Prevalence and impact of unhealthy weight in a national sample of us adolescents with autism and other learning and behavioral disabilities. Maternal Child Health J..

[8] Zuckerman K., Hill A., Guion K., Voltolina L., Fombonne E. (2014). Overweight and obesity: Prevalence and correlates in a large clinical sample of children with autism spectrum disorder. J. Autism Dev. Disord..

[9] Ahearn W.H., Castine T., Nault K., Green G. (2001). An assessment of food acceptance in children with autism or pervasive developmental disorder-not otherwise specified. J. Autism Dev. Dis..

[10] Bandini L.G., Gleason J., Curtin C., Lividini K., Anderson S.E., Cermak S.A., Must A. (2013). Comparison of physical activity between children with autism spectrum disorders and typically developing children. Autism.

[11] Schreck K.A., Williams K. (2006). Food preferences and factors influencing food selectivity for children with autism spectrum disorders. Res. Dev. Disabil..

[12] Schreck K.A., Williams K., Smith A.F. (2004). A comparison of eating behaviors between children with and without autism. J. Autism Dev. Disord..

[13] Must A., Phillips S., Bandini L. (2005). Longitudinal fruit and vegetable consumption, fiber, and glycemic load as predictors of fatness and relative weight change over adolescence in girls. Obes. Res..

[14] Gase L.N., Robles B., Barragan N.C., Kuo T. (2014). Relationship between nutritional knowledge and the amount of sugar-sweetened beverages consumed in los Angeles county. Health Educ. Behav..

[15] Ludwig D.S., Peterson K.E., Gortmaker S.L. (2001). Relation between consumption of sugar-sweetened drinks and childhood obesity: A prospective, observational analysis. Lancet.

[16] Must A., Tybor D.J. (2005). Physical activity and sedentary behavior: A review of longitudinal studies of weight and adiposity in youth. Int. J. Obes..

[17] Tam C.S., Garnett S.P., Cowell C.T., Campbell K., Cabrera G., Baur L.A. (2006). Soft drink consumption and excess weight gain in Australian school students: Results from the Nepean study. Int. J. Obes..

[18] Rimmer J.H., Rubin S.S., Braddock D. (2000). Barriers to exercise in African American women with physical disabilities. Arch. Phys. Med. Rehab..

[19] Rimmer J.H., Riley B., Wang E., Rauworth A., Jurkowski J. (2004). Physical activity participation among persons with disabilities: Barriers and facilitators. Am. J. Prev. Med..

[20] Rimmer J.H., Rowland J.L., Yamaki K. (2007). Obesity and secondary conditions in adolescents with disabilities: Addressing the needs of an underserved population. J. Adolesc. Health.

[21] Bryant E.S., Duncan M.J., Birch S.L. (2014). Fundamental movement skills and weight status in British primary school children. Eur. J. Sport Sci..

[22] Cliff D.P., Okely A.D., Smith L.M., McKeen K. (2009). Relationships between fundamental movement skills and objectively measured physical activity in preschool children. Pediatr. Exerc. Sci..

[23] Erwin H.E., Castelli D.M. (2008). National physical education standards: A summary of student performance and its correlates. Res. Q. Exerc. Sport.

[24] Bandini L.G., Anderson S.E., Curtin C., Cermak S., Evans E.W., Scampini R., Must A. (2010). Food selectivity in children with autism spectrum disorders and typically developing children. J. Pediatr..

[25] Obrusnikova I., Cavalier A. (2011). Perceived barriers and facilitators of participation in after-school physical activity by children with autism spectrum disorders. J. Dev. Phys. Disabil..

[26] Rimmer J.H., Rowland J.L. (2008). Health promotion for people with disabilities: Implications for empowering the person and promoting disability-friendly environments. Am. J. Lifes. Med..

[27] Liu T., Hamilton M., Davis L., ElGarhy S. (2014). Gross motor performance by children with autism spectrum disorder and typically developing children on TGMD-2. Child Adolesc. Behav..

[28] Pan C., Tsai C., Chu C. (2009). Fundamental movement skills in children diagnosed with autism spectrum disorders and attention deficit hyperactivity disorder. J. Autism Dev. Disord..

[29] Provost B., Heimerl S., Lopez B. (2007). Levels of gross and fine motor development in young children with autism spectrum disorder. Phys. Occup. Ther. Pediat..

[30] Staples K., Reid G. (2010). Fundamental movement skills and autism spectrum disorders. J. Autism Dev. Disord..

[31] Sallis J.F., Glanz K. (2006). The role of built environments in physical activity, eating, and obesity in childhood. Future Child..

[32] Must A., Parisi S.M. (2009). Sedentary behavior and sleep: Paradoxical effects in association with childhood obesity. Int. J. Obes..

[33] Must A., Phillips S.M., Curtin C., Anderson S.E., Maslin M., Lividini K., Bandini L.G. (2014). Comparison of sedentary behaviors between children with autism spectrum disorders and typically developing children. Autism Int. J. Res. Pract..

[34] Elbaum B., Vaughn S. (1999). Can school-based interventions enhance the self-concept of students with learning disabilities?. Except. Parent.

[35] Griffiths L.J., Parsons T.J., Hill A.J. (2010). Self-esteem and quality of life in obese children and adolescents: A systematic review. Int. J. Pediat. Obes..

[36] Simpson C.G., Swicegood P.R., Gaus M.D. (2006). Nutrition and fitness curriculum: Designing instructional interventions for children with developmental disabilities. Teach. Except. Child..

[37] Henderson S.E., Sugden D.A., Barnett A.L., Brown T., Lalor A. (2009). Movement assessment battery for children—Second edition. Phys. Occup. Ther. Pediat..

[38] Carlson A., Rowe E., Curby T. (2013). Disentangling fine motor skills’ relations to academic achievement: The relative contributions of visual-spatial integration and visual-motor coordination. J. Genet. Psychol..

[39] Dinehart L., Manfra L. (2013). Associations between low-income children’s fine motor skills in preschool and academic performance in second grade. Early Educ. Dev..

[40] Francis M., Dalrymple N., Nichols S.S.D. (2010). The effects of a school-based intervention program on dietary intakes and physical activity among primary-school children in Trinidad and Tobago. Public Health Nutr..

[41] Klohe-Lehman D., Freeland-Graves J., Anderson E.R., McDowell T., Clarke K.K., Hanss-Nuss H., Milani T.J. (2006). Nutrition knowledge is associated with greater weight loss in obese and overweight low-income mothers. J. Am. Dietet. Assoc..

[42] Poh B.K., Kathryn Tham B.L., Wong S.N., Winnie Chee S.S., Tee E.S. (2012). Nutritional status, dietary intake patterns and nutrition knowledge of children aged 5–6 years attending kindergartens in the klang valley, Malaysia. Malays. J. Nutr..

[43] Triches R.M., Giugliani E. (2005). Obesity, eating habits and nutritional knowledge among school children. Revista de Saude Publica.

[44] Wardle J., Parmenter K., Waller J. (2000). Nutrition knowledge and food intake. Appetite.

[45] Jobling A. (2001). Beyond sex and cooking: Health education for individuals with intellectual disability. Mental Retard..

[46] Curtin C., Jojic M., Bandini L.G. (2014). Obesity in children with autism spectrum disorder. Harv. Rev. Psychiat..

[47] Guo S.S., Wu W., Chumlea W.C., Roche A.F. (2002). Predicting overweight and obesity in adulthood from body mass index values in childhood and adolescence. Am. J. Clin. Nutr..

[48] Must A., Strauss R.S. (1999). Risks and consequences of childhood and adolescent obesity. Int. J. Obes. Relat. Metab. Disord..

[49] Tyler C.V., Schramm S.C., Karafa M., Tang A.S., Jain A.K. (2011). Chronic disease risks in young adults with autism spectrum disorder: Forewarned is forearmed. Am. J. Intell. Dev. Disab..

[50] Centers for Disease Control and Prevention Data and statistics. https://www.cdc.gov/ncbddd/autism/data.html.

[51] American Psychiatric Association (2013). Diagnostic and Statistical Manual of Mental Disorders.

[52] Gower J.R., Slater H., Jordan K.C., Moyer-Mileur L., Wilkinson R.D. (2010). Validity and reliability of a nutrition knowledge survey for assessment in elementary school children. J. Am. Dietet. Assoc..

[53] Kuczmarski R.J., Ogden C.L., Guo S.S., Grummer-Strawn L., Flegal K.M., Mei Z., Johnson C.L. (2002). 2000 CDC growth charts for the United States: Methods and development. Vital Health Stat..

[54] Krebs N.F., Himes J.H., Jacobson D., Nicklas T.A., Guilday P., Styne D. (2007). Assessment of child and adolescent overweight and obesity. Pediatrics.

[55] Graf C., Koch B., Kretschmann-Kandel E., Falkowski G., Christ H., Coburger S., Dordel S. (2004). Correlation between BMI, leisure habits and motor abilities in childhood (CHILT-project). Int. J. Obes. Relat. Metab. Disord..

[56] Greier K., Riechelmann H., Burtscher M. (2014). Prevalence of Obesity and Motor Performance Capabilities in Tyrolean Preschool Children.

[57] Osmani A., Driton M. (2014). Differences in the motoric abilities of students due to the body mass index (BMI). Sport Mont..

[58] D’Hondt E., Deforche B., Gentier I., Verstuyf J., Vaeyens R., Bourdeaudhuij I., Lenoir M. (2014). A longitudinal study of gross motor coordination and weight status in children. Obesity.

[59] Frey G.C., Chow B. (2006). Relationship between BMI, physical fitness, and motor skills in youth with mild intellectual disabilities. Int. J. Obes..

[60] Pitetti K.H., Yarmer D.A., Fernhall B. (2001). Cardiovascular fitness and body composition of youth with and without mental retardation. Adapt. Phys. Activ. Q..

[61] Burns C.M., Richman R., Caterson I.D. (1987). Nutrition knowledge in the obese and overweight. Int. J. Obes..

[62] Muehler C., Hobbs J., Lipira P. (2006). Is body mass index (BMI) related to nutrition knowledge in children?. Mo. J. Health Phys. Educ. Recr. Dance.

[63] Reinehr T., Kersting M., Chahda C., Andler W. (2003). Nutritional knowledge of obese and nonobese children. Nutr. Res..

[64] Thakur N., D’Amico F. (1999). Relationship of nutrition knowledge and obesity in adolescence. Family Med..

[65] Stankiewicz M., Pieszko M., Sliwinska A., Malgorzewicz S., Wierucki L., Zdrojewski T., Lysiak-Szydlowska W. (2014). Obesity and diet awareness among polish children and adolescents in small towns and villages. Cent. Eur. J. Public Health.

[66] Srinivasan S.M., Pescatello L.S., Bhat A.N. (2014). Current perspectives on physical activity and exercise recommendations for children and adolescents with autism spectrum disorders. Phys. Ther..

[67] Alkon A., Crowley A.A., Benjamin Neelon S.E., Hill S., Pan Y., Nguyen V., Kotch J.B. (2014). Nutrition and physical activity randomized control trial in child care centers improves knowledge, policies, and children’s body mass index. BMC Public Health.

[68] Hinckson E.A., Dickinson A., Water T., Sands M., Penman L. (2013). Physical activity, dietary habits and overall health in overweight and obese children and youth with intellectual disability or autism. Res. Dev. Disabil..

[69] Yu O.K.C., Rhee Y.K.C., Sohn H.S.C., Cha Y.S.C. (2010). Effects of nutrition education on overweight and obese children in Chonbuk area—Focus on food habit, eating behaviors, dietary attitude, nutrition knowledge and nutrients intake. J. Korean Soc. Food Sci. Nutr..

[70] Davison K.K., Birch L.L. (2001). Childhood overweight: A contextual model and recommendations for future research. Obes. Rev..

[71] Cermak S.A., Bandini L.G., Curtin C. (2010). Food selectivity and sensory sensitivity in children with autism spectrum disorders. J. Am. Diet. Assoc..

[72] Kohn M., Rees J.M., Brill S., Fonseca H., Jacobson M., Katzman D., Schneider M. (2006). Preventing and treating adolescent obesity: A position paper of the society for adolescent medicine. J. Adolesc. Health.

